# Status on Genetic Resistance to Rice Blast Disease in the Post-Genomic Era

**DOI:** 10.3390/plants14050807

**Published:** 2025-03-05

**Authors:** Rodrigo Pedrozo, Aron Osakina, Yixiao Huang, Camila Primieri Nicolli, Li Wang, Yulin Jia

**Affiliations:** 1 USDA ARS Dale Bumpers National Rice Research Center, Stuttgart, AR 72160, USA; rodrigo.pedrozo@usda.gov (R.P.); aron.osakina@usda.gov (A.O.); yixiao.huang@usda.gov (Y.H.); li.wang@usda.gov (L.W.); 2 Department of Biology, Washington University in St. Louis, St. Louis, MO 63130, USA; 3 Entomology and Plant Pathology Department, University of Arkansas, Rice Research and Extension Center (RREC), Stuttgart, AR 72160, USA; cnicolli@uada.edu

**Keywords:** rice (*Oryza sativa* L.), plant disease resistance, rice blast, *Magnaporthe oryzae* (syn. *Pyricularia oryzae*), AI-based tools

## Abstract

Rice blast, caused by *Magnaporthe oryzae*, is a major threat to global rice production, necessitating the development of resistant cultivars through genetic improvement. Breakthroughs in rice genomics, including the complete genome sequencing of *japonica* and *indica* subspecies and the availability of various sequence-based molecular markers, have greatly advanced the genetic analysis of blast resistance. To date, approximately 122 blast-resistance genes have been identified, with 39 of these genes cloned and molecularly characterized. The application of these findings in marker-assisted selection (MAS) has significantly improved rice breeding, allowing for the efficient integration of multiple resistance genes into elite cultivars, enhancing both the durability and spectrum of resistance. Pangenomic studies, along with AI-driven tools like AlphaFold2, RoseTTAFold, and AlphaFold3, have further accelerated the identification and functional characterization of resistance genes, expediting the breeding process. Future rice blast disease management will depend on leveraging these advanced genomic and computational technologies. Emphasis should be placed on enhancing computational tools for the large-scale screening of resistance genes and utilizing gene editing technologies such as CRISPR-Cas9 for functional validation and targeted resistance enhancement and deployment. These approaches will be crucial for advancing rice blast resistance, ensuring food security, and promoting agricultural sustainability.

## 1. Introduction

### 1.1. Background and Economical Relevance of Blast Disease for Rice Growing Regions

Rice (*Oryza sativa* L.) is one of the most important foods for half of the world’s population. *Oryza sativa* is directly consumed by humans and livestock and is used in countless byproducts. One of the most significant constraints for stable rice production globally is rice blast disease caused by the fungus *Magnaporthe oryzae* (Hebert) Barr.

**Figure 1:**
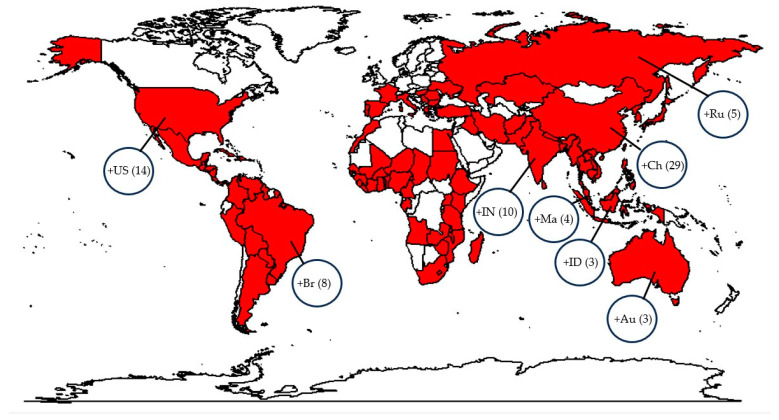
Worldwide distribution of rice blast disease. Red color shows a total of 101 countries and regions where blast disease has been reported according to the most recent CABI distribution maps of plant diseases, in compliance with EPPO [*Magnaporthe oryzae* (rice blast disease)|CABI Compendium (cabidigitallibrary.org)]. (+Country(No)) represents subnational records of *Magnaporthe oryzae* per country; (+Ch(28))China: Anhui, Chongqing, Fujian, Gansu, Guangdong, Guangxi, Guizhou, Hainan, Hebei, Heilongjiang, Henan, Hong Kong, Hubei, Hunan, Inner Mongolia, Jiangsu, Jiangxi, Jilin, Liaoning, Ningxia, Qinghai, Shaanxi, Shandong, Shanghai, Shanxi, Sichuan, Xinjiang, Yunnan, Zhejiang; (+IN(9))India: Andhra Pradesh, Delhi, Himachal Pradesh, Jammu and Kashmir, Karnataka, Kerala, Tamil Nadu, Uttar Pradesh, Uttarakhand; (+ID(2))Indonesia: Java, Lesser Sunda Islands; (+Ma(3))Malaysia: Peninsular Malaysia, Sabah, Sarawa; (+Ru2)Russia: Russia (Europe), Russian Far East; (+US(14))United States: Alabama, Arkansas, Delaware, Florida, Georgia, Hawaii, Kentucky, Louisiana, Maryland, Mississippi, Missouri, Ohio, Oregon, Texas; (+Au(3))Australia: Northern Territory, Queensland, Western Australia; (+Br(8))Brazil: Distrito Federal, Espirito Santo, Goias, Mato Grosso, Mato Grosso do Sul, Minas Gerais, Para, Parana, Pernambuco, Piaui, Rio Grande do Sul, Rondonia, Santa Catarina, Sao Paulo, Tocantins. The full dataset with further information can be found in the Supplementary Materials (Table S1) [5].

There is substantial evidence that the early domestication of *O. sativa* from its wild relative *O. rufipogon* in the middle Yangtze valley in China occurred about 7000 years before present (BP) [1,2]. Centuries of rice production globally in over 100 countries have made rice an excellent habitat for microorganisms, such as the filamentous fungus *M. oryzae*, the causal agent of rice blast disease. *M. oryzae* from Italian millet (*Setaria italica*) expanded to rice between 7000 BP in China and 3000 BP in Korea and Japan [3,4], spreading to the rest of the world’s rice production areas subsequently. Currently, rice blast is widely distributed around the globe, with 101 countries/regions reporting the presence of the causal agent (Figure 1).

*M. oryzae* is taxonomically different from *M. grisea*, a species complex of haploid ascomycetous fungi [6]. Hosts for the *M. grisea* species complex include several weeds in rice paddies such as torpedo grass (*Panicum repens*) and cutgrass (*Leersia hexandra*), as well as *S. italica* [7]. Host specificity is generally observed among most hosts in nature; however, under controlled conditions, cross-infection may occur between a grass and rice [8].

Despite significant advancements in genomics, breeding, and disease management strategies, rice blast disease (*Magnaporthe oryzae*) remains a persistent threat to global rice production. The pathogen’s high genetic variability and rapid adaptation to resistance genes undermine the durability of resistant rice cultivars, leading to recurrent outbreaks and yield losses ranging from 10% to 30% annually. The global economic impact is substantial, with estimated losses exceeding 157 million tons of rice per year. Additionally, the increasing unpredictability of climate change exacerbates disease severity by creating favorable conditions for pathogen proliferation. Current management approaches, including chemical control, resistant varieties, and cultural practices, are often ineffective in the long term due to the pathogen’s ability to overcome host resistance and environmental variability. Thus, a more integrative and adaptive approach that combines genomic innovations and precision breeding is necessary to develop sustainable and broad-spectrum resistance against rice blast disease [9].

### 1.2. The Biology of Magnaporthe oryzae

*Magnaporthe oryzae* is one of the most devastating diseases affecting rice (*Oryza sativa* L.) production worldwide. This pathogen is known for its ability to infect all above-ground parts of the rice plant, leading to significant yield losses under favorable conditions. The biology of *M. oryzae* and its interaction with the rice host have been extensively studied to understand its infection mechanisms, symptom development, and the genetic basis of resistance. The disease has two commonly recognized phases: leaf blast and neck blast (syn. panicle blast).

**Figure 2:**
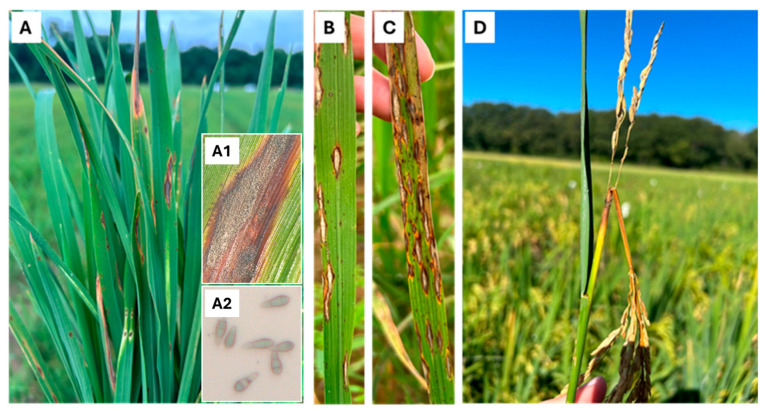
Characteristic symptoms of rice blast disease caused by *Magnaporthe oryzae*. (**A**–**C**) show typical diamond-shaped lesions on rice leaves, indicative of leaf blast. (**A1**,**A2**) display *M. oryzae* sporulation on blast lesions, highlighting fungal development on the leaf surface. (**D**) illustrates symptoms of neck blast, where infection at the panicle base leads to tissue necrosis, weakening the stem and potentially causing grain sterility and yield loss.

For the first phase, initial infection in young seedlings begins when conidia (asexual spores) are deposited on leaf surfaces. Water is essential for spore germination and attachment to the leaf surface [10,11]. Under optimal conditions, spore germination occurs rapidly, forming polarized germ tubes within hours [12]. Infection occurs through a specialized structure of the fungus named the appressorium. The appressorium generates high turgor pressure, allowing it to penetrate the leaf cuticle and cell wall [13,14]. Secondary infection cycles are initiated by spores produced from lesions on young seedlings, with thousands of spores produced from a single lesion within about 15 days post-infection. These lesions are typically diamond-shaped, initially appearing dark green or gray with brown borders, and later becoming light tan with necrotic borders (Figure 2A,A1,B). Under favorable conditions, the lesions can merge and enlarge, potentially killing the leaf and ultimately leading to plant death (Figure 2C,D). Resistant cultivars, however, typically exhibit small brown to dark brown lesions.

The severity of rice blast and spore production per lesion (Figure 2A2) depends on several factors, including temperature, field conditions, relative humidity, fertility levels, and the genotype of rice cultivars. Moderate temperatures (~24 °C), high relative humidity (90–92%), and high moisture levels with at least a 12 h wetness period are conducive to the disease. The severity of the disease during the vegetative phase significantly influences the disease’s impact during the reproductive phase. In the second phase, spores produced at the end of the growing season can cause neck blast, often resulting in direct crop loss due to its consequent infection of panicles. Yield reduction inflicted by neck blast infection is significantly more severe than leaf blast, with losses approaching 70%, or even 100%, of the anticipated yield under epidemic conditions [15].

Over the past century, the extensive analysis of the rice germplasm has revealed that major blast resistance (*R*) genes (*Piricular*, *Pi*-genes) confer complete genetic resistance (vertical resistance). These genes are often specific to strains of *M. oryzae* containing the corresponding avirulence (*AVR*) genes. Incomplete resistance (horizontal resistance) is typically conditioned by multiple genes located on different chromosomal regions, known as quantitative trait loci (QTLs). Germplasms carrying both major and minor *R* genes are crucial genetic resources for rice breeders aiming to enhance blast resistance in elite rice varieties.

Understanding the biology of *M. oryzae* and the genetic basis of resistance in rice is essential for developing effective management strategies against rice blast disease. Ongoing research and breeding programs continue to focus on identifying and utilizing resistant germplasms to mitigate the impact of this pathogen on rice production, considering that new virulent strains of the pathogen will always emerge a few years after new *R* genes are deployed that can ‘defeat’ the existing resistance.

## 2. Mapped Blast *R* Genes

### 2.1. Current Status

The discovery and use of disease resistance genes have been central to managing rice blast disease. Advances in genetic mapping technologies, including RFLP, SSRs, and SNPs, have enabled the identification and precise localization of numerous blast resistance genes on rice chromosomes [9]. Access to complete genome sequences of rice subspecies indica and japonica (http://rgp.dna.affrc.go.jp, accessed on 17 January 2025) has further enhanced fine-scale mapping, supporting the development of broad-spectrum-resistant rice varieties. Resistance to *M. oryzae* is mediated by *R* genes and is based on the gene-for-gene interaction concept [16]. This resistance occurs when the products of the *R* gene specifically recognize the corresponding *M. oryzae* effector and activate effector-triggered immunity (ETI) [17]. To date, it is estimated that about 122 *R* genes/alleles to rice blast have been identified, most of them from *O. sativa* japonica and indica cultivars and a minority from wild species of rice. They are distributed among all 12 rice chromosomes and a great majority are located on chromosomes 6, 11, and 12, which harbor 23, 29, and 25 resistance genes, respectively. Chromosomes 3 and 7 contain the lowest number of blast-resistant genes, with only one gene noted so far in each (*Pi66(t)* and Pi17(t), respectively) (Table S2).

Classical and molecular genetic analyses have revealed that a great majority of blast *R* genes are distributed in clusters consisting of allelic or tightly linked genes. Three major clusters of blast-resistance genes have been detected in rice on chromosomes 6, 11, and 12, while others are distributed on chromosomes 1, 2, 4, 5, 8, and 9 [18]. For instance, the blast *R* genes *Pi8*, *Pi9*, *Pi26(t)*, *Pi27(t)*, *Pi40*, *Piz*, *Pi-2*, *Piz-t*, *Pigm(t)*, and *Pi59(t)* have been mapped at the *Piz* locus on chromosome 6 [19], and at least nine *R* genes have been mapped to the *R* gene cluster on the telomeric end of rice chromosome 11 where, seven of them (*Pik*, *Pi-ks*, *Pikp*, *Pikm*, *Pikh*, *Pik1*, and *Pi1*) have been shown to be alleles of the *Pik* locus [20,21,22]. Similarly, *Pi12*, *Pita*, *Pita2*, *Pi39(t)*, *Ptr*, *Pi42*, *Pi24*, *Pi20*, *PiGD3*, and *Pi157* have been mapped to the *R* gene cluster at the centromeric region of chromosome 12 (Table 1) [23,24,25,26]. Several of blast *R* genes have been cloned to date, such as *Pi36* [27].

In fact, *Pib* from the Tohoku IL9 rice variety considered the first blast resistant gene to be cloned and the latest being *Pijx*, which confers broad-spectrum resistance to early and late plant physiological stage blast [4]. Besides the *Pijx* gene, other cloned genes such as *Ptr* [26], *Pigm* [79], Pi1 [21], *Pi5* [80], *Pi7* [81], *Pi9* [82], *Pi21* [83], *Pi50* [84], *Pi54* [85], *Pi57* [86], and *Pi64* [87] were also reported to confer broad-spectrum resistance against rice blast. Furthermore, other well-known cloned *R* genes that also confer broad-spectrum resistance include *Pita* [27], *Pib* [88], and *Pizh* [89], while cloned genes with partial resistance include *Pi21* [83] and *Pb1* [90].

Partial resistance is usually not race-specific and can offer durable resistance, although it is not as commonly efficient as major genes. This resistance is mediated by minor *R* genes (or QTLs). In this review we are presenting 17 well-known minor blast *R* genes that have been identified (Table 2).

Although is known that more than 700 QTLs have been discovered over recent decades [100]. Achieving durable resistance requires leveraging race-nonspecific, broad-spectrum genes by characterizing quantitative trait loci (QTLs) governing blast resistance. While numerous QTLs have been reported in diverse genetic backgrounds and environments, their deployment in marker-assisted selection (MAS) is often hindered by environmental variability, genetic background effects, and challenges in bi-parental mapping populations. Despite these limitations, QTL mapping remains critical for understanding the genetic basis of resistance traits.

Meta-QTL analysis is a powerful tool for improving resistance breeding in rice. This method combines data from multiple independent studies to refine QTL positions, reduce confidence intervals, and improve candidate gene identification [100]. Traditional QTL mapping often faces challenges such as broad confidence intervals, environmental sensitivity, and linkage drag. Meta-QTL analysis addresses these issues by consolidating QTL information into consensus maps, allowing for more precise and stable gene discovery.

By narrowing QTL intervals and identifying consensus regions with greater accuracy, this approach accelerates the identification and deployment of *R* genes with broad-spectrum resistance. As a result, breeders can develop next-generation rice varieties that are better equipped to withstand evolving pathogen populations and diverse environmental conditions.

Despite significant progress in identifying and characterizing genes that confer resistance to leaf blast, genetic research on neck blast resistance has not advanced as rapidly. Neck blast, historically more economically damaging than leaf blast, can lead to yield losses of 70–100% under epidemic conditions [15]. Previous studies [15,23,101,102] have suggested that this discrepancy may stem from intrinsic differences in the resistance mechanisms between leaf and neck blast. Discrepancies in the mapping locations of QTLs associated with resistance to leaf and neck blast have implied the existence of distinct genetic pathways for resistance to these diseases. However, there are instances where genes or QTLs associated with panicle blast resistance have been mapped to the same genomic regions as major leaf blast resistance genes in the rice genome. This suggests the possibility of common genes conferring resistance to both leaf and neck blast [103].

This hypothesis is further supported by studies indicating that the expression pattern of a blast *R* gene is a critical factor in determining whether it will confer protection against both leaf and neck blast. The genes *Pb1* and *Pi64*, identified for adult-stage panicle blast resistance from the indica cultivar ‘Modan’, serve as examples of how expression patterns can influence disease resistance. The *Pb1* gene, located on the long arm of chromosome 11, encodes a coiled coil–nucleotide-binding site–leucine-rich repeat (CC–NBS–LRR) protein [90,104]. Introduced into several elite rice varieties commercially grown in Japan, *Pb1* has maintained resistance for nearly 30 years without signs of breakdown [90]. The gene shows low expression during early vegetative stages but gradually increases, reaching peak levels at full heading. Remarkably, Nipponbare, a susceptible cultivar transformed to overexpress *Pb1*, displayed strong resistance to leaf blast during early vegetative stages [90]. Similarly, Ma et al. [87] identified the *Pi64* gene from the broad-spectrum-resistant japonica landrace Yangmaogu (YMG). Located on chromosome 1, *Pi64* encodes a CC–NBS–LRR protein and is constitutively expressed throughout all developmental stages and tissues examined. This consistent expression pattern is believed to be a key factor in its effectiveness against both leaf and neck blast. It is suggested that most *R* genes exhibit constitutive expression, apart from *Pi21*, *Pi5*, *Pi63*, and *Pb1*, which are only induced upon pathogen attack [105].

These findings highlight that the expression pattern of a blast *R* gene is, indeed, a key factor in determining its effectiveness in protecting against leaf blast, neck blast, or both disease stages. Genes that are predominantly active during the early vegetative phase tend to confer resistance to leaf blast. In contrast, those expressed during the flag leaf and heading stages are more likely to protect against neck and panicle blast. Furthermore, genes that are consistently expressed throughout the plant are expected to provide resistance across both disease phases. These conclusions support earlier research suggesting the existence of common genes that resist both leaf and neck blast [102,103,106].

### 2.2. Weedy Rice as a Novel Source of Blast Resistance

Among the 122 blast *R* genes identified so far, only a small proportion (about 4%) have been derived from wild rice relatives. Given that many beneficial alleles may have been left behind during rice domestication and evolution, these untapped genetic resources warrant further exploration to identify new genes for blast resistance. Weedy rice, an agricultural pest that competes with cultivated rice, contains novel *R* genes that are a potential target for blast disease resistance [107,108]. Furthermore, new blast QTLs against blast resistance have been identified in US weedy rice species [109]. Researchers should continue exploring new allelic variants of already known and unknown resistance genes from weedy rice, unexplored landraces, and wild species. For example, using sequencing allele mining to uncover new alleles with diverse resistance specificities, promoter region mining may uncover variants with differing expression patterns.

### 2.3. Pyramiding Multiple R Genes Enhance Broad Spectrum and Durable Resistance

The deployment of *R* genes in commercial varieties through breeding programs is the most effective and economical strategy for managing rice blast disease. However, the frequent loss of *R* gene-mediated resistance has been reported [110,111,112], representing a significant challenge to breeding programs regarding the long-term use of single *R* genes. Pyramiding, the practice of combining multiple *R* genes with overlapped resistance spectra in a single cultivar, is an attractive option and can be the most suitable way to achieve durable and broad-spectrum resistance. The rice variety Jefferson, for instance, which contains a combination of two *R* genes (*Pik*/*Piz*), has remained resistant since its first application in 1997 [63,113].

Several studies on the combination (pyramiding) of blast *R* genes have been reported. Chen et al. [114] showed that pyramiding *Pi1* and *Pi2* increases resistance to 98.04% against 715 isolates, compared to monogenic lines with *Pi1* and *Pi2*, which have 45% and 89.65% resistance, respectively [114]. Similarly, the resistance spectrum of pyramiding *Pi-ta* and *Pi46* is enhanced compared to the monogenic lines *Pi46* and *Pita* [115]. Furthermore, Yu et al. showed that pyramiding two different genes with overlapping resistance spectra can improve the resistance of plants [116]. In addition, a combination of *Pi21*, *Pi34*, *qBR4-2*, and *qBR12-1* together offers superior resistance compared to their monogenic lines [117].

However, although the main objective of pyramiding *R* genes is to achieve a broad spectrum of resistance, instances of reduced resistance due to gene pyramiding have also been confirmed. For instance, the resistance from the *Pi2*/*Pita* combination was reported to be lower compared to lines carrying only *Pi2* [118]. In another similar study, a significantly lowered resistance was observed in *Pi9* and *Pi54* combination lines when compared to the monogenic lines harboring *Pi9* [119]. These studies showed that combining *R* genes does not always directly result in an elevated resistance response. Therefore, to achieve broad-spectrum resistance, the *R* gene combination patterns should be thoroughly assessed before incorporating targeted *R* genes into breeding programs.

## 3. Structure and Function of Blast *R* Genes

The molecular characterization and mapping of blast *R* genes in rice have significantly advanced our understanding and capabilities in breeding for disease resistance. Among the major blast *R* genes, 39 have been molecularly characterized, including *Pit*, *Pish*, *Pi35*, *Pi37*, *Pi64*, *Pib*, *Pi21*, *Pi63*, *PiPR1*, *Pi2*, *Pizt*, *Pi9*, *Pi7h*, *Pigm*, *Pi50*, *Pid2*, *Pid3*, *Pid3*-*A4*, *Pi25*, *Pi36*, *Pi5*, *Pi56*, *Pii*, *Pib3*, *Pik*, *Pi1*, *Pikm*, *Pik-h*, *Pik-p*, *Pike*, *Pi-CO39*, *Pi54* (Tetep), *Pi54of* (*Oryza officinalis*), *Pi54rb*, *Pia*, *Pb1*, *Pi65*, *Ptr*, *Pijx*, and *Pita* (Table 3).

Most of these genes encode proteins with the nucleotide-binding site (NBS) and leucine-rich repeat (LRR) domains characteristic of the largest class of plant *R* genes [21,27,90,123,134]. Notably, the *Pid2* gene encodes a serine–threonine kinase membrane-spanning protein [128], while *Pi21* encodes a protein with heavy-metal binding and proline-rich domains [83], and *Ptr* encodes a putative E3 ligase with four Armadillo repeats [26].

The structural similarities of blast R proteins, characterized by NBS and LRR domains, suggest that conserved regions play functional roles in resistance. These proteins can be divided into two subgroups: TIR-NBS-LRR, containing a Toll-interleukin receptor-like domain, and CC-NBS-LRR, with a putative coiled-coil structure. The rice genome comprises about 500 NBS-LRR gene families, mostly belonging to the CC-NBS-LRR family. The NBS domain, containing kinase motifs, binds ATP or GTP and triggers downstream signal transduction, while the LRR domain recognizes pathogen effectors [21,27,87,140,141].

Most cloned blast *R* genes provide complete resistance to strains of *M. oryzae* that contain the corresponding *AVR* genes. For some avirulent races, multiple members of the same gene family, such as *Pi5*, *Pik*, *Pikp*, *Pikm*, and *Pia*, are required for complete resistance. Regardless, the genetic mapping and molecular cloning of different blast *R* genes have provided an array of gene-linked, gene-based, or functional markers for the efficient selection of resistance genes in breeding programs [90,129,142]. Structural comparisons of the cloned members of multi-allelic resistance loci have also provided information on the DNA regions within these genes responsible for their distinct resistance specificities and offered the possibility of using these as the basis for identifying different resistance alleles to be used in newly developed varieties [20,21,125].

## 4. The *AVR* Gene in *M. oryzae*

When *M. oryzae* enter the host cell, a variety of effectors are secreted into plant cells. These effectors can modulate plant immunity and facilitate infection. Functional proteins are the biggest class of effectors for plant pathogenic fungi [143]. Some effectors can be recognized by resistance (R) proteins and these effectors are called avirulence (AVR) proteins. The efficacy of major *R* genes is determined by their cognate *AVR* genes. So far, 14 *AVR* genes from *M. oryzae* have been cloned (Table 4), including ToxB-like effector gene *AVR-Pi54*, *AVR-Pita*, *AVR-Pizt*, *AVR1-CO39*, and *AVR-Pik/km/kp*; six cysteine residue-containing effector *AVR-Pi9*; putative polyketide synthase and nonribosomal peptide synthetase (PKS/NRPS) effector *ACE1*; zinc metalloprotease effector *AVR-Pita*; glycine-rich effectors *PWL1* and *PWL2*; zinc finger transcription factors *MoHTR1* and *MoHTR2*; and two other effectors, *AVR-Pib* and *AVR-Pii*, which do not contain typical known structures [105,143].

*AVR-Pita* at the telomeric region of chromosome 3 has a neutral zinc metalloprotease at its N-terminus, which is thought to be recognized by *Pi-ta* to trigger immunity response [144]. *AVR-Pita* can interact with the cytochrome c oxidase (COX) assembly protein OsCOX11, a regulator of reactive oxygen species (ROS) in host mitochondria. Overexpressing *AVR-Pita* decreases ROS accumulation in rice [157]. *AVR-Pi54* on chromosome 3 has a 19 amino acid long SP at its N-terminal. The predicted mature protein of *AVR-Pi54* shows that the mature protein has six β -sheets but no α-helices [145]. Reports have shown that *AVR-Pi54* can translocate to the nucleus and plasma membrane of plant cells when *Pi54* is absent. However, when both *Pi54* and *AVR-Pi54* are present, they interact at the plasma membrane only, and not in the nucleus [158]. *AVR-Pi9* is a gene located in the middle of chromosome 7. It encodes a small, secreted protein which can translocate into biotrophic interfacial complex (BIC) during infection and the mature protein has six cysteine residues and two LxAR-like motifs. In addition, *AVR-Pi9* can affect the stability of RING-type E3 ubiquitin ligase OsRGLG5 in rice, which positively regulates plant resistance against rice blast [158]. The resistance gene *Pia*’s cognate gene *AVR-Pia* encodes a small, secreted protein (88 amino acids) and it is in either chromosome 5 or 7. It is in a region prone to chromosome rearrangement by MAGGY transposons [147]. Two NBS-LRR proteins, RGA4 and RGA5, can also interact with *AVR-Pi9* to mediate resistance against rice blast [159]. *AVR-Pib* is located at chromosome 3 and encodes a small protein with a signal peptide of 22 amino acids at the N terminal [148]. *AVR-Pib* can interact with SH3P2, an SH3 domain-containing protein, which can also interact with Pia but has a lower affinity [160]. *AVR-Pii* is a 70-amino-acid-long protein with an N-terminal signal peptide [147]. *AVR-Pii* contains a Zinc-finger motif in its C terminal, and it binds to a conserved hydrophobic pocket in Exo70. The interaction of *AVR-Pii* and Exo70 is important for *Pii*-mediated resistance [161]. *AVR-Pik/km/kp* is in chromosome 1 and encodes a 113-amino-acid protein with an N terminal signal peptide [147,162]. *AVR-Pik* interacts with the heavy-metal-associated (HMA) domain of *Pik-1* to induce plant immunity [163]. *AVR-Pik* can also bind other HMA containing proteins such as OsHIPP19 and OsHIPP20 to affect plant immunity [164]. *M. oryzae* Host Transcription Reprogramming 1 and 2 or *MoHtr1* and *MoHtr2* are two cytoplasmic effectors containing a C2H2 zinc finger domain. They are secreted via BIC and can translocate to nuclei of plant cells to affect the expression of immunity-associated genes [153]. *Pizt*’s cognate gene *AVR-Pizt* is a cytoplasmic effector gene located in chromosome 7. It can suppress programed cell death triggered by proapoptotic protein BAX in *Nicotiana benthamiana*, suggesting that *AVR-Pizt* may contribute to the virulence of *M. oryzae* by inhibiting the plant immunity response [149]. AVR-*Pizt* can also interact with RING-type E3 ligases AVRPIZ-T INTERACTING PROTEIN 10 (APIP10), APIP6, and bZIP transcription factor APIP5 to adjust host immunity response [165,166,167]. The resistant gene *Pi33*’s cognate gene *ACE1* was mapped to chromosome 8 and encodes a 4035-amino-acid-long protein, which is a hybrid protein between a polyketide synthase (PKS) and a nonribosomal peptide synthetase (NRPS). Both PKS domains and NRPS domains were found in *ACE1* [150]. The mutation of one amino acid in the catalytic site of the β-ketoacyl synthase domain in *ACE1* abolishes its recognition by resistant plants, suggesting that the enzyme activity is required for protein function [150]. *AVR-CO39* was mapped to chromosome 1 and encodes a small, secreted protein with a predicted signal peptide in its N-terminus. *AVR-CO39* is expressed during infection and can translocate into the cytoplasm of rice cells [135]. Two NBS-LRR genes, *RGA4* and *RGA5*, are required for *AVR-CO39*-mediated resistance, and one isoform of RGA5, RGA5-A, can directly interact with *AVR-CO39* [168]. *PW1* and *PW2* are two small glycine-rich effectors located in chromosome 2. Four genes, *PW1–PW4*, were found in the effector family, but only *PW1* and *PW2* contribute to the pathogenesis of *M. oryzae* [151,152]. *PW2* is induced at the biotrophic stage of fungal infection, and its expression requires some tandem repeats in the promoter region [169].

## 5. Management of Blast Disease Using Marker-Assisted Selection (MAS)

To date, 122 blast *R* genes have been mapped with closely linked DNA markers [8,107]. The implementation of MAS in rice breeding programs has revolutionized the development of blast-resistant rice varieties. By leveraging molecular markers linked to blast *R* genes, MAS accelerates the breeding process, allowing breeders to select desired traits at an earlier stage of development with greater precision and efficiency. This approach facilitates the pyramiding of multiple *R* genes, enhancing the durability and effectiveness of resistance against a broad spectrum of blast strains. As a result, MAS has significantly improved the efficiency and effectiveness of breeding programs, leading to the development of rice varieties with predictable and stable levels of blast resistance across diverse growing conditions. Overall, MAS has played a crucial role in addressing the challenges posed by blast disease in rice cultivation, contributing to increased agricultural productivity, food security, and sustainability.

Traditionally, the integration of major blast *R* genes has been achieved through classical breeding methods, a process that can be expedited through the utilization of marker-assisted selection (MAS) [170].MAS serves as a powerful tool in the management of rice blast disease. This innovative technique enables breeders to identify and select plants with genetic markers linked to resistance against the blast pathogen. MAS expedites the breeding process by allowing for the direct selection of desired traits without the need for time-consuming and labor-intensive field evaluations [171]. Through the identification of specific genetic markers associated with resistance to blast disease, breeders can efficiently screen large populations of rice plants to pinpoint those with the highest resistance potential.

The evolution of MAS has significantly advanced rice breeding, particularly in addressing blast disease resistance. Early efforts were focused on developing DNA markers from cloned *R* genes like *Pita* and *Pib*, crucial for blast resistance, with notable contributions from Jia et al. [142] in identifying markers for the *Pita* gene, pivotal for blast resistance in the United States. Subsequent advancements led to the identification of linked markers for four other blast *R* genes effective against multiple *M. oryzae* races. MAS facilitated the incorporation of key blast *R* genes like *Pi1*, *Pi5*, *Piz-5*, and *Pita* into diverse rice genotypes, as highlighted by Jia in 2009. Notably, the identification of the *Pi66(t)* gene, mapped to chromosome 11, stands out as significant. To support breeders, novel PCR-based molecular markers—WRKY41, NBS-LRR-970-1, and NBS-LRR-970-2—were developed for the *Pi66(t)* locus [172]. Validation studies demonstrated the efficacy of WRKY41 and NBS-LRR-970-1 in segregating with *Pi66(t)* in recombinant inbred line (RIL) populations and their utility as *Pi66(t)*-linked specific markers in USDA–ARS rice mini-core collections. These breakthrough markers not only streamline the identification of resistant lines with *Pi66(t)* in mapping populations, but also facilitate the screening of diverse rice germplasms.

Marker-assisted selection (MAS) has been widely applied in rice breeding programs outside the United States, especially in regions like India, China, and Southeast Asia, where rice is a staple crop, but its application is not limited to these areas. In 2018, Mushk Budji, renowned for its aroma and quality but susceptible to blast disease, was crossed with the triple-gene donor line DHMAS 70Q 164-1b. Through MAS across first and second backcross generations, blast resistance genes *Pi54*, *Pi1*, and *Pita* were incorporated, resulting in improved lines exhibiting resistance under both artificial and natural field conditions in India [173]. Similarly, in Taiwan, marker-assisted backcrossing was employed to develop monogenic lines with diverse blast resistance genes in the elite japonica rice cultivar Kaohsiung 145 (KH145).

In 2020, a group from India [174] undertook a study to develop high-yielding climate-resilient rice, using genes conferring resistance against blast (*Pi9*), bacterial leaf blight (BLB) (*Xa4*, *xa5*, *xa13*, *Xa21*), brown planthopper (BPH) (Bph3, Bph17), gall midge (GM) (Gm4, Gm8), and QTLs for drought tolerance (qDTY1.1 and qDTY3.1) through marker-assisted forward breeding (MAFB). In the same year, Tian et al. developed a new insertion/deletion (InDel) marker, *Pigm/2/9InDel*, that can differentiate the cloned *R* genes (*Pigm*, *Pi9*, and *Pi2/Piz-t*) at the *Pi2/9* locus, indicating that *Pigm* and *Pi2* alleles were introgressed for blast resistance breeding, mainly in the Fujian and Guangdong region, and that *Pi9* is a valuable blast resistance resource to be introduced into China.

A year later, a group from Taiwan, utilizing blast-resistant lines from the International Rice Research Institute (IRRI) as resistance donors, carried out genetic analysis and subsequent backcrossing, yielding 1,499 homozygous resistant lines evaluated for their resistance and agronomic performance, with promising lines showing potential for durable resistance in field cultivation [175]. In Bangladesh, another study with DNA markers closely linked to blast-resistant genes, including *Pb1*, *pi21*, and *Piz*, were utilized in foreground selection to develop advanced lines with superior morphological and pathogenicity performance compared to the recurrent parent, suggesting their potential as donors or varieties resistant to rice blast disease management [176]. In 2022, the breeding potential of the *Pigm* gene was thoroughly assessed in the context of geng/japonica rice breeding in Jiangsu province [177]. By utilizing backcrossing and marker-assisted selection (MAS), the *Pigm* gene was incorporated into two rice cultivars, Wuyungeng 32 (WYG32) and Huageng 8 (HG8). For each genetic background, five advanced backcross lines (ABLs) containing the *Pigm* gene were developed, maintaining the same genotypes as the corresponding recurrent parent for the other 13 known *R* gene loci. When compared to their respective recurrent parents, all ABLs demonstrated significantly enhanced resistance in seedling inoculation assays. Additionally, in terms of panicle blast resistance, all ABLs achieved high resistance levels to blast disease across tests conducted over three consecutive years, involving inoculation with seven mixed conidial suspensions from various regions of Jiangsu province. These findings indicate that the *Pigm* gene holds considerable breeding value for developing rice varieties that exhibit durable and broad-spectrum resistance to blast disease and the success of using MAS. Moreover, in Italy, marker-assisted back crossing (MABC) demonstrated efficacy in pyramiding blast-resistance (*Pi*) genes into an elite japonica rice variety, resulting in broad-spectrum resistance against multivirulent strains [178]. These findings underscore the utility of marker-assisted breeding approaches in bolstering rice blast disease management and enhancing the resilience of rice varieties against this devastating pathogen.

## 6. Using Computational Power and Artificial Intelligence (AI) for Unraveling Molecular Mechanism of Rice Blast Disease Resistance

Recent advancements in sequencing technologies and computational approaches have paved the way for pangenomic studies, which offer a more comprehensive understanding of the genomic landscape [179].

The concept of pangenome initially referred to the full complement of genes from a species divided into the core genome, consisting of genes present in all individuals, and the dispensable genome, in which genes are not present in all individuals and some may be unique to individuals [180]. With increasing computational power, we can now construct graph pangenomes that include all of the genetic variations in a species, such as SNPs, Indel, and structure variations using computational pipelines such as the PanGenome Graph Builder (PGGB) or Minigraph-Cactus [181,182]. For example, a pangenome of *Magnaporthe oryzae* with 156 isolates identified 24,100 genes, nearly double that of the reference genome 70-15 [183].

Pangenomics has proven particularly valuable in capturing the extensive genetic diversity found in crops like rice, which have undergone domestication and breeding processes that have shaped their genomes. By analyzing multiple de novo-assembled genomes, pangenomics can identify novel genes, structural variations, and presence/absence variations that are missed when relying solely on a single reference genome. Studies have constructed rice pangenomes from hundreds to thousands of accessions [184,185,186,187]. A key finding is that a substantial fraction of genes, often over 50%, are not shared across all rice lines. Genes overlapping structural variants frequently show relatively low expression levels compared with non-affected genes [186]. This highlights how structural changes in the genome can have broad impacts on gene regulation and phenotypic diversity. Pangenomics has also enabled tracing the evolutionary origins and fates of gene families important for agronomic traits like disease resistance. For example, Shang et al. [188] systematically analyzed the NBS-LRRs genes across the rice pangenome, showing that most *NLR* genes in cultivated varieties were retained from their wild progenitor species. While unlocking this comprehensive view of genomic diversity, however, it also poses challenges in terms of data analysis, computational requirements, and the need for robust experimental validation [179].

**Figure 3:**
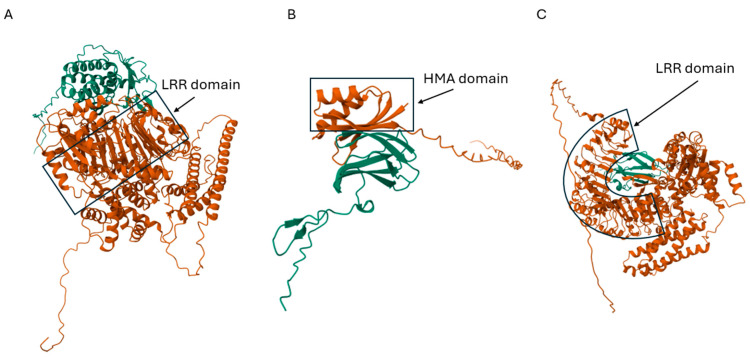
Prediction of the interaction structures of *M*. *oryzae AVR* genes with rice *R* genes using Alphafold2 and AlphaFold3. AVR-Pita interaction with Pi-ta through leucine-rich repeat (LRR) domain (**A**), AVR-Pik recognition of OsHIPP19 through heavy metal-associated (HMA) domain (**B**), and AVR-Pik interaction with Pi9 through two nearby LRR domains (**C**).

In parallel, recent advances in artificial-intelligence-based protein structure prediction tools like AlphaFold2, RoseTTAFold, and AlphaFold3 hold great promise for accelerating the identification and functional validation of new resistance genes [189,190,191,192]. For example, Wang et al. [193], using a pan-gene analysis using three *M. oryzae* isolates from different races IB49, IC17, and IE1K, showed that 92% of 11,015 non-redundant genes are core genes, with 926 non-redundant genes specific to different races. Additionally, in the same study, it was shown that 84% of predicted effectors were identified as core genes, with 43 of them specific to different races, highlighting the pathogen’s adaptability and specialization. The authors further predicted the interaction structures of *M. oryzae AVR* genes with rice *R* genes using Alphafold2 and AlphaFold3. The analysis showed that AVR-Pita can interact with Pi-ta through the leucine-rich repeat (LRR) domain (Figure 3A), AVR-Pik recognizes OsHIPP19 through the heavy metal-associated (HMA) domain (Figure 3B), and AVR-Pik can also interacts with Pi9 through two nearby LRR domains (Figure 3C). Thus, these findings enhance our understanding of the plant–pathogen interaction mechanisms, which could significantly aid rice blast resistant breeding programs.

The pangenome provides a comprehensive view of the genetic diversity within rice, uncovering all possible *R* genes and alleles across different cultivars [188] and the genetic diversity of rice blast pathogens, uncovering all possible effectors and alleles across different isolates [193]. By analyzing this extensive genetic repertoire, researchers can identify key *R* genes that may be absent or underrepresented in local rice cultivars and key effectors that are probably more popular within local isolates but not in the isolates from other locations. AI-based protein structure prediction tools provide a powerful approach to predict the role of these resistance genes and their interactions with the rice blast pathogens, offering deeper insights into the molecular mechanisms of resistance.

Finally, gene editing techniques like CRISPR-Cas9 enable the precise manipulation of these resistance genes, allowing researchers to validate their functions and introduce beneficial alleles into rice cultivars. This integrated approach not only accelerates the identification of resistance genetic resources, but also facilitates the development of elite rice varieties with enhanced, durable resistance to local rice blast isolates.

One key example is the *Bsr*-d1 gene, a negative transcription factor linked to broad-spectrum blast resistance [194]. Studies have shown that the blast-resistant allele of OsBsr-d1 is absent in most japonica rice varieties in Northeast China. Using CRISPR/Cas9, researchers successfully knocked out Bsr-d1 in the japonica rice variety Jigeng88, resulting in mutant lines (KO#1 and KO#2) with significantly enhanced resistance to multiple *Magnaporthe oryzae* races. These mutants exhibited increased hydrogen peroxide production upon infection, which likely contributed to their improved defense response. Transcriptomic analysis further revealed the upregulation of genes involved in defense-related pathways, supporting the role of *Bsr*-d1 knockout in strengthening rice blast resistance.

Beyond *Bsr*-d1, CRISPR/Cas9 has been employed to simultaneously enhance resistance against multiple diseases. For instance, the editing of Pi21 [195], a susceptibility gene, along with modifications to the effector-binding element (EBE) of OsSULTR3;6, conferred dual resistance to both rice blast and bacterial leaf streak. The resulting mutants showed significantly reduced lesion areas for both diseases without compromising agronomic traits, demonstrating the potential of multiplex genome editing in rice improvement.

The integration of CRISPR/Cas9 into rice breeding pipelines offers a powerful strategy for developing next-generation rice cultivars with durable, broad-spectrum resistance. By precisely targeting susceptibility genes and enhancing resistance mechanisms, genome editing accelerates the improvement of elite rice varieties while reducing dependence on chemical fungicides. As new studies continue to expand the applications of CRISPR-based approaches, the post-genomic era presents unprecedented opportunities for sustainable and resilient rice production.

## 7. Conclusions and Future Directions

Harnessing disease *R* genes to breed rice varieties with broad-spectrum resistance is a pivotal approach to controlling rice blast disease caused by *Magnaporthe oryzae*. In recent years, the post-genomic era has ushered in remarkable advancements in our understanding and management of this disease. The complete sequencing of the genomes of japonica and indica rice subspecies, coupled with the availability of a vast array of molecular markers, has significantly advanced our capacity to perform a detailed genetic analysis of blast resistance in rice.

These breakthroughs have resulted in the identification of approximately 122 blast-resistance genes, 39 of which have been successfully cloned and characterized at the molecular level. The genetic mapping and cloning of these genes have produced a rich collection of tightly linked or functional markers, essential tools for marker-assisted selection (MAS) in rice breeding. By leveraging these markers, MAS has transformed rice breeding practices, enabling the precise incorporation of multiple resistance genes into high-yielding cultivars, thereby strengthening both the durability and breadth of resistance.

Moreover, the rise of pangenomics and the deployment of advanced artificial intelligence tools such as AlphaFold2, RoseTTAFold, and AlphaFold3 have greatly accelerated the identification and functional analysis of resistance genes. These technologies not only streamline the breeding process, but also provide profound insights into the genetic and structural mechanisms underpinning resistance, thereby supporting the development of rice varieties with robust, long-lasting resistance to blast disease.

As we look to the future, the effective management of rice blast disease will increasingly rely on the integration of innovative genomic and computational techniques. Future research should prioritize the refinement of AI-based tools for the large-scale screening of *R* genes and their interactions with pathogen effectors, which will enhance the functional annotation and validation of novel *R* genes. Additionally, the application of functional genomics and gene-editing technologies, such as CRISPR-Cas9, will be essential for confirming the roles of candidate *R* genes and for engineering targeted modifications to boost resistance. By advancing these strategies, we can continue to enhance rice blast resistance, contributing to global food security and sustainable agricultural practices.

## Figures and Tables

**Table 1:** List of the major *R* genes and their chromosomal location, map position, linked markers, and donor variety.

No.	*R* Gene Name	Chr. No. ^1^	Genomic Region (Mb) ^2^	Linked Marker	Donor(s)	Ref. ^3^
1	*Pitp(t)*	1	25.13	RFLP, SNP	Tetep	[28]
2	*Pi27(t)*	1	5.55	SSR	Q14	[29]
3	*Pi-h2(t)*	1	25.13	SSR	HR4	[30]
4	*Pi14(t)*	2	34.94	RFLP	Maowangu	[31]
5	*PiDa(t)*	2	2.21	SSR	Dacca6	[32]
6	*Pid1(t)*	2	34.94	SSR, RFLP	Digu	[33]
7	*Piy(t)*	2	35.03	SSR	Yanxian No. 1	[34]
8	*Pig(t)*	2	34.34	SSR	Guangchangzhan	[35]
9	*Pitq-5*	2	34.61	RFLP	Teqing	[36]
10	*Pi16(t)*	2	34.94	RFLP	Aus373	[37]
11	*Pi39(t)*	4	32.68	SSR	Chubu 111	[38]
12	*Pi10*	4	14.52	RAPD	Tongil	[39]
13	*Pias(t)*	4	31.26	SSR, CAPS	Asominori	[40]
14	*Pi23(t)*	5	10.75	RFLP, SSR	Suweon 365	[41]
15	*Pi2-1*	6	10.08	SSR, SFP	Tianjingyeshengdao	[42]
16	*Pi2-2*	6	10.20	SSR	Jefferson	[43]
17	*Pi8(t)*	6	11.36	-	Kasalath	[44]
18	*Pi13(t)*	6	15.83	RFLP	Maowangu	[31]
19	*Pi13(t)*	6	15.83	SSR	Kasalath	[45]
20	*Pi22(t)*	6	4.89	RFLP	Suweon 365	[41]
21	Pi25 *	6	12.33	RFLP, RGA, SSR	Gumei 2	[46]
22	*Pi26(t)*	6	11.06	RFLP, SSR	Gumei 2	[47]
23	*Pi40(t)*	6	9.86	STS, SSR	R65482-4-136-2-2 *O. australiensis*	[48]
24	*Pi50(t)*	6	10.41	SSR, CAPS	Er-Ba-Zhan	[49]
25	*Piz*	6	-	-	Zenith	[50]
26	*Pitq-1*	6	-	RFLP	Teqing	[36]
27	*Pid4*	6	-	-	Digu	[51]
28	*Pi17(t)*	7	22.25	Isozyme	DJ123	[52]
29	*Pi33(t)*	8	7.56	SSR, RFLP	IR64	[53]
30	*Pi55(t)*	8	25.58	SSR, STS	Yuejingsimiao 2	[54]
31	*PiGD-1(t)*	8	16.37	SSR, RFLP, RGA	Sanhuangzhan 2	[24]
32	*Pi15(t)*	9	9.61	SSR, CRG	GA25	[55]
33	*Pi56(t)*	9	9.77	SSR, CRG, SNP	Sanhuangzhan 2	[56]
34	*PiGD-2(t)*	10	-	SSR, RFLP, RGA	Sanhuangzhan 2	[24]
35	*Pi7(t)*	11	18.64	RFLP	Moroberekan	[57]
36	*Pi18(t)*	11	28.93	RFLP	Suweon 365	[58]
37	*Pi38(t)*	11	22.48	SSR, AFLP	Tadukan	[59]
38	*Pi43(t)*	11	27.67	SSR	Zhe733	[60]
39	*Pi44(t)*	11	28.93	RFLP, STS, AFLP	Moroberekan	[61]
40	*Pi47*	11	27.67	SSR	Xiangzi 3150	[62]
41	*Pilm-2*	11	28.93	RFLP	Lemont	[36]
42	*Piks*	11	27.31	SSR	Bengal, M201	[63]
43	*Pikg(t)*	11	-	-	GA20	[64]
44	*Piy(t)*	11	-	RM202	Yunyin	[65]
45	*Pi60(t)*	11	-	InDel, SSR	93-11	[66]
46	*Pizy(t)*	11	-	RM206	Ziyu44	[67]
47	*Pi1*	11	28.00	RFLP	C101LAC	[21]
48	*Pi-h1*	11		SSR	HR4	[30]
49	*Pi6(t)*	12	7.73	RFLP	Apura	[68]
50	*Pi19(t)*	12	10.73	SSR	Aichi Asahi	[69]
51	*Pi12(t)*	12	7.73	RFLP	Hong Jiao Zhan	[70]
52	*Pi20(t)*	12	12.95	SSR	IR24	[71]
53	Pi24(t) *	12	10.60	RFLP, RAPD, RGA	Zhong 156	[23]
54	*Pi39(t)*	12	10.61	SSR	Q15	[72]
55	*Pi41(t)*	12	16.74	SSR, STS	93-11	[73]
56	*Pi48*	12	11.95	SSR	Xiangzi 3150	[62]
57	*Pi57(t)*	12	12.37	RFLP	Moroberekan	[74]
58	*Pita 3(t)*	12	9.89	SSR	IRBLta2-Re	[75]
59	*Pih3(t)*	12	12.95	SSR	HR4	[30]
60	*Pi51(t)*	12	11.95	SSR, SFP	Tianjingyeshengdao	[42]
61	*Pi61(t)*	12	9.98	InDel, SSR	93-11	[66]
62	*Pi62(t)*	12	7.73	RAPD, RFLP	Yashiro-mochi	[76]
63	*Pitq-6*	12	7.73	RFLP	Teqing	[73]
64	*Pitb*	12	9.37	SSR, InDel	ZixuanDonor(s) variety	[77]
65	*Pi67*	12	12.09	SSR	Tetep	[78]
66	*PiGD*-*3(t)*	12	14.45	SSR, RFLP	Sanhuangzhan 2	[24]

^1^ Rice chromosome number; ^2^ mega-base (Mb); ^3^ references. * This *R* gene shares its name with another known *R* gene.

**Table 2:** List of selected minor *R* genes and their chromosomal location, genomic region, linked markers, and donor variety.

No.	*R* Gene Names	Chr. No. ^1^	Genomic Region (Mb) ^2^	Linked Marker	Donor(s)	Ref. ^3^
1	*Pi35(t)*	1	32.1	SSR	Hokkai 188	[91]
2	*Pir2-3*	2	-	SSR	IR64	[92]
3	*Pirf2-1(t)*	2	-	SSR	*O. rufipogon*	[92]
4	*Pi66(t)*	3	26.78	SSR	AS20-1	[93]
5	Pi24(t) *	1	-	-	Azucena	[94]
6	*Pikur1*	4	-	Isozyme	Kuroka	[94]
7	*Pikahei1(t)*	4	31.67	SSR, SNP	Kahei	[95]
8	*Pi26(t)*	5	2.78	RFLP, RAPD,	Azucena	[96]
9	*Pi27(t)*	6	6.92	RFLP	IR64	[96]
10	*Pi11(t)*	8	13.93	RFLP, RAPD	Zhaiyeqing	[97]
11	*Pi29(t)*	8	13.93	RFLP, RAPD, Isozyme	IR64	[96]
12	*Pi28(t)*	10	21.04	RFLP, RAPD	Azucena	[96]
13	*Pif*	11	-	-	Chugoku 31-1 (St. No. 1)	[98]
14	*Pi30(t)*	11	4.41	RFLP, RAPD, Isozyme	IR64	[96]
15	*Pi31(t)*	12	11.93	RFLP, RAPD	IR64	[96]
16	*Pi32(t)*	12	21.24	RFLP, RAPD	IR64	[96]
17	*Pi34(t)*	12	-	-	Chubu32	[99]

^1^ Rice chromosome number; ^2^ mega-base (Mb); ^3^ references. * This *R* gene shares its name with another known *R* gene.

**Table 3:** List of the blast *R* genes cloned and their chromosomal location, genomic region, encoding protein, and donor variety.

No.	*R* Gene Names	Chr. No. ^1^	Genomic Region (Mb) ^2^	Encoding Protein	Donor(s)	Ref. ^3^
1	*Pit*	1	2.27	CC-NBS-LRR	K29	[120]
2	*Pish*	1	33.3	NBS-LRR	Nipponbare	[121]
3	*Pi35*	1	32.1	NBS-LRR	Hokkai 188	[91]
4	*Pi37*	1	33.1	NBS-LRR	St. NO. 1	[122]
5	*Pi64*	1	32.31	NBS-LRR	Yangmaogu	[87]
6	*Pib*	2	35.10	NBS-LRR	Tohuku IL9	[88]
7	*Pi21*	4	19.81	Proline-rich metal Binding protein	Owarihatamochi	[83]
8	*Pi63*	4	13.05	NBS-LRR	Kahei	[123]
9	*PiPR1*	4	-	NBS-LRR	Unknown	[124]
10	*Pi2*	6	10.39	NBS-LRR	Jefferson	[125]
11	*Pizt*	6	10.39	NBS-LRR	zenit	[125]
12	*Pi9*	6	10.38	NBS-LRR	75-1-127	[126]
13	*Pizh*	6	-	NBS-LRR		[89]
14	*Pigm*	6	10.36	NBS-LRR	Gumei4	[127]
15	*Pid2*	6	17.16	B-lectin receptor Kinase	Digu	[128]
16	*Pid3*	6	13.05	NBS-LRR	Digu	[129]
17	*Pid3-A4*	6	-	NBS-LRR	*Oryza rufipogon*	[130]
18	*Pi25*	6	12.33	NBS-LRR	Gumei2	[46]
19	*Pi36*	8	2.87	NBS-LRR	Kasalath	[27]
20	*Pi5*	9	9.77	NBS-LRR	RIL260	[80]
21	*Pi56*	9	9.77	NBS-LRR	Sanhuangzha	[126]
22	*Pii*	9	-	NBS-LRR	Hitomebore	[89]
23	*Pb3*	11	-	NBS-LRR	Dongjin	[131]
24	*Pik*	11	28.01	NBS-LRR	Kusabue	[20]
25	*Pi1*	11	-	NBS-LRR	C101LAC	[21]
26	*Pikm*	11	28.00	NBS-LRR	Tsuyuake	[132]
27	*Pik-h*	11	24.99	NBS-LRR	K3	[130]
28	*Pik-p*	11	28.05	NBS-LRR	K60	[133]
29	*Pike*	11	28.00	NBS-LRR	Xiangzao143	[134]
30	*Pi-CO39*	11	6.66	NBS-LRR	CO39	[135]
31	*Pi54*	11	25.26	NBS-LRR	Tetep	[85]
32	*Pi54*	11	25.26	NBS-LRR	*Oryza. officinalis*	[136]
33	*Pi54rh*	11	25.26	NBS-LRR	*Oryza. rhizomatis*	[131]
34	*Pia*	11	6.49	NBS-LRR	Sasanishiki	[137]
35	*Pb1*	11	-	NBS-LRR	Modan	[90]
36	*Pi-ta*	12	10.60	NBS-LRR	Yashiro-mochi	[27]
37	*Pi65*	12	-	LRR-RLK	GangYu129	[138]
38	*Ptr*	12	10.8	ARM repeat	Katy	[26]
39	*Pijx*	12	-	NBS-LRR	-	[139]

^1^ Rice chromosome number; ^2^ mega-base (Mb); ^3^ references.

**Table 4:** List of the blast *AVR* genes cloned and their chromosomal location, genomic region, effector type, and cognate *R* gene.

*AVR* Gene	Protein Size	Chr. No. ^1^	Effector Type	Cognate *R* Gene	Ref. ^2^
*AVR-Pita*	223	3	Zinc-dependent Metalloprotease	*Pi-ta*	[144]
*AVR-Pi54*	153	4	MAX-effectors *	*Pi54, Pi54rh, Pi54of*	[145]
*AVR-Pi9*	91	7	Six cysteine residues and two LxAR-like motifs	*Pi9*	[146]
*AVR-Pia*	85	5 or 7	MAX-effectors *	*Pia*	[147]
*AVR-Pib*	75	3	MAX-effectors *	*Pib*	[148]
*AVR-Pii*	70	7	Zink-finger effector fold	*Pii*	[147]
*AVR-Pik/km/kp*	113	1	MAX-effectors *	*Pik/Pik-m/Pik-p, Pik-h*	[147]
*AVR-Pizt*	108	7	MAX-effectors *	*Piz-t*	[149]
*ACE1*	4035	1	PKS/NRPS	*Pi33*	[150]
*AVR1-CO39*	89	1	MAX-effectors *	*Pi-CO39*	[135]
*PWL1*	147	2	Glycine-rich	Unknown	[151]
*PWL2*	145	2	Glycine-rich	Unknown	[152]
*MoHTR1*	198	Unknown	Zincfinger transcription factor	Unknown	[153]
*MoHTR2*	110	7	Zincfinger transcription factor	Unknown	[153]

^1^ *Magnaporthe oryzae* chromosome number; ^2^ references. * MAX-effectors (*Magnaporthe* Avrs and *ToxB*-like) were defined by the 3D structure of proteins [143,154,155,156].

## Supplementary Materials

The following supporting information can be downloaded at https://www.mdpi.com/article/10.3390/plants14050807/s1: Table S1: Global spread of rice blast disease. All 102 affected countries based on latest CABI distribution maps and EPPO compliance are listed. Table S2: Distribution of major, minor, and cloned *R* genes to blast among the 12 rice chromosomes.
