# Supplementary material for: Status on Genetic Resistance to Rice Blast Disease in the Post-Genomic Era
Source: Plants (Basel). 2025 Mar 5;14(5):807. doi: 10.3390/plants14050807 (PMC11901910; doi:10.3390/plants14050807)
Supplement: Supplementary file 1 [file plants-14-00807-s001.zip › plants-3458770-supplementary.pdf]

Supplementary materials_TS1-2

Table S1. Global spread of rice blast disease. All the 101 affected countries/regions based on latest CABI distribution maps and EPPO compliance is listed.

Country (Region)	State (Sub-region)	Latitude	Longitude	Presence	Extent[1]
Angola		-12.5	18.5	Present	Not recorded
Benin		9.5	2.25	Present	Not recorded
Burkina Faso		12.5	-1.66667	Present	Not recorded
Burundi		-3.5	30	Present	Not recorded
Cabo Verde		16	-24	Present	Not recorded
Cameroon		6	12.5	Present	Not recorded
Chad		15	19	Present	Not recorded
Congo, Democratic Republic of the		-2.5	23.5	Present	Not recorded
Ivory Coast		8	-5.5	Present	Not recorded
Egypt		27	30	Present	Not recorded
Ethiopia		9	39.5	Present	Not recorded
Gabon		-1	11.75	Present	Not recorded
Gambia		13.5	-15.5	Present	Not recorded
Ghana		8.1	-1.2	Present	Not recorded
Guinea		10.83333	-10.6667	Present	Not recorded
Kenya		1	38	Present	Not recorded
Liberia		6.5	-9.5	Present	Localized
Madagascar		-20	47	Present	Not recorded
Malawi		-13.5	34	Present	Not recorded
Mali		18	-2	Present	Not recorded
Mauritius		-20.3	57.58333	Present	Not recorded
Morocco		32	-6	Present	Not recorded
Mozambique		-18.25	35	Present	Not recorded
Niger		18	9	Present	Not recorded
Nigeria		10	8	Present	Not recorded
Senegal		14.5	-14.25	Present	Not recorded
Sierra Leone		8.5	-11.5	Present	Not recorded
South Africa		-29	24	Present	Not recorded
Sudan		16	30	Present	Not recorded
Tanzania		-6	35	Present	Not recorded
Togo		8.66667	1.08333	Present	Not recorded
Uganda		1.25	32.5	Present	Not recorded
Zambia		-14.3333	28.5	Present	Not recorded
Zimbabwe		-19	29.75	Present	Not recorded
Afghanistan		33	66	Present	Not recorded
Bangladesh		24	90	Present	Widespread

Brunei		4.5	114.6667	Present	Not recorded
Cambodia		13	105	Present	Not recorded
China		35	105	Present	Localized
	- Anhui	31.91667	117.1667	Present	Not recorded
	- Chongqing	30.08333	107.8333	Present	Not recorded
	- Fujian	26.25	118	Present	Not recorded
	- Gansu	37.75	102.75	Present	Not recorded
	- Guangdong	23.5	113.25	Present	Not recorded
	- Guangxi	24	109	Present	Not recorded
	- Guizhou	27	107	Present	Not recorded
	- Hainan	19.25	109.75	Present	Not recorded
	- Hebei	39	115.6667	Present	Not recorded
	- Heilongjiang	47.75	128	Present	Not recorded
	- Henan	34	113.6667	Present	Not recorded
	- Hubei	31	112.25	Present	Not recorded
	- Hunan	27.66667	111.75	Present	Not recorded
	- Inner Mongolia	43.5	114.75	Present	Not recorded
	- Jiangsu	33	119.8333	Present	Not recorded
	- Jiangxi	27.66667	115.6667	Present	Not recorded
	- Jilin	43.58333	126.1667	Present	Not recorded
	- Liaoning	41.25	122.6667	Present	Not recorded
	- Ningxia	37.25	106	Present	Not recorded
	- Qinghai	36	96	Present	Not recorded
	- Shaanxi	36	109	Present	Not recorded
	- Shandong	36.33333	118.25	Present	Not recorded
	- Shanghai	31.16667	121.4167	Present	Not recorded
	- Shanxi	37.66667	112.25	Present	Not recorded
	- Sichuan	30.5	102.6667	Present	Not recorded
	- Xinjiang	41.5	85.5	Present	Not recorded
	- Yunnan	25	101.5	Present	Not recorded
	- Zhejiang	29.16667	120	Present	Not recorded
	-Hong Kong	22.25	114.1667	Present	Not recorded
India		22	79	Present	Widespread
	- Andhra Pradesh	15.83333	79.75	Present	Not recorded
	- Delhi	28.6667	77.1	Present	Not recorded
	- Himachal Pradesh	31.91667	77.25	Present	Not recorded
	- Jammu and Kashmir	33.91667	76.66667	Present	Not recorded
	- Karnataka	14.66667	75.83333	Present	Not recorded
	- Kerala	10.41667	76.5	Present	Not recorded
	- Tamil Nadu	11	78.33333	Present	Not recorded
	- Uttar Pradesh	27.25	80.75	Present	Not recorded
	- Uttarakhand	30.25	79.25	Present	Not recorded
Indonesia		-5	120	Present	Not recorded
	- Java	-7.49167	110.0044	Present	Not recorded

	- Lesser Sunda Islands	-9	120	Present	Not recorded
Iran		32	53	Present	Not recorded
Iraq		33	44	Present	Not recorded
Japan		35.68536	139.7531	Present	Widespread
Kazakhstan		48	68	Present	Not recorded
Laos		18	105	Present	Widespread
Malaysia		2.5	112.5	Present	Widespread
	- Peninsular Malaysia	4	102	Present	Not recorded
	- Sabah	5.5	117	Present	Not recorded
	- Sarawak	2.5	113.5	Present	Not recorded
Myanmar		21	96	Present	Not recorded
Nepal		28	84	Present	Not recorded
North Korea		40	127	Present	Not recorded
Pakistan		30	70	Present	Not recorded
Philippines		13	122	Present	Not recorded
South Korea		36.5	127.75	Present	Not recorded
Sri Lanka		7.75	80.75	Present	Not recorded
Taiwan		24	121	Present	Not recorded
Thailand		15.5	101	Present	Not recorded
Turkey		39	35	Present	Not recorded
Vietnam		16.16667	107.8333	Present	Not recorded
Bulgaria		42.66667	25.25	Present	Widespread
France		46	2	Present	Not recorded
Greece		39	22	Present	Not recorded
Hungary		47	20	Present	Widespread
Italy		42.83333	12.83333	Present	Not recorded
Portugal		39.6945	-8.13057	Present	Widespread
Romania		46	25	Present	Not recorded
Russia		60	100	Present	Localized
	- Russia (Europe)	59.25	45.62	Present	Not recorded
	- Russian Far East	53.1	134.88	Present	Not recorded
Serbia		44.81892	20.45998	Present	Not recorded
Spain		40	-4	Present	Not recorded
Ukraine		49	32	Present	Not recorded
Belize		17.25	-88.75	Present	Not recorded
Bermuda		32.33022	-64.74	Present	Not recorded
Costa Rica		10	-84	Present	Not recorded
Cuba		22	-79.5	Present	Not recorded
Dominican Republic		19	-70.6667	Present	Not recorded
El Salvador		13.83333	-88.9167	Present	Not recorded
Guatemala		15.5	-90.25	Present	Not recorded
Haiti		19.07582	-72.2962	Present	Not recorded
Honduras		15	-86.5	Present	Not recorded
Martinique		14.66667	-61	Present	Not recorded

Mexico		23	-102	Present	Not recorded
Nicaragua		13	-85	Present	Not recorded
Panama		9	-80	Present	Not recorded
Puerto Rico		18.24829	-66.4999	Present	Not recorded
Trinidad and Tobago		11	-61	Present	Not recorded
United States		39.76	-98.5	Present	Localized
	- Alabama	32.75041	-86.7503	Present	Not recorded
	- Arkansas	34.75037	-92.5004	Present	Not recorded
	- Delaware	39.00039	-75.4999	Present	Not recorded
	- Florida	28.75054	-82.5001	Present	Not recorded
	- Georgia	32.75042	-83.5002	Present	Not recorded
	- Hawaii	20.78785	-156.386	Present	Not recorded
	- Kentucky	38.20042	-84.8776	Present	Not recorded
	- Louisiana	31.00047	-92.0004	Present	Not recorded
	- Maryland	39.00039	-76.75	Present	Not recorded
	- Mississippi	32.75041	-89.7504	Present	Not recorded
	- Missouri	38.25031	-92.5005	Present	Not recorded
	- Ohio	40.25034	-83.0002	Present	Not recorded
	- Oregon	44.00013	-120.501	Present	Not recorded
	- Texas	31.25044	-99.2506	Present	Not recorded
American Samoa		-14.2338	-169.478	Present	Not recorded
Australia		-25	135	Present	Localized
	- Northern Territory	-20	134	Present	Not recorded
	- Queensland	-20	145	Present	Not recorded
	- Western Australia	-25	122	Present	Not recorded
Federated States of Micronesia		6.924	158.162	Present	Not recorded
Fiji		-18	178	Present	Not recorded
New Caledonia		-21.5	165.5	Present	Not recorded
Argentina		-34	-64	Present	Not recorded
Bolivia		-17	-65	Present	Not recorded
Brazil		-10	-55	Present	Widespread
	- Distrito Federal	-15.75	-47.75	Present	Not recorded
	- Espirito Santo	-20	-40.75	Present	Not recorded
	- Goias	-15.5807	-49.6362	Present	Not recorded
	- Mato Grosso	-13	-56	Present	Not recorded
	- Mato Grosso do Sul	-20.5	-55	Present	Not recorded
	- Minas Gerais	-18	-44	Present	Not recorded
	- Para	-4	-53	Present	Not recorded
	- Parana	-24.5	-51.3333	Present	Not recorded
	- Pernambuco	-8.33333	-37.75	Present	Not recorded
	- Piaui	-7.25	-42.3333	Present	Not recorded
	- Rio Grande do Sul	-30	-53.5	Present	Not recorded
	- Rondonia	-11	-63	Present	Not recorded
	- Santa Catarina	-27	-50	Present	Not recorded

	- Sao Paulo	-22	-49	Present	Not recorded
	- Tocantins	-10.5	-48	Present	Not recorded
Colombia		4	-73.25	Present	Not recorded
French Guiana		4	-53	Present	Not recorded
Guyana		5	-59	Present	Not recorded
Paraguay		-23.3333	-58	Present	Not recorded
Peru		-10	-75.25	Present	Not recorded
Uruguay		-33	-56	Present	Not recorded
Venezuela		8	-66	Present	Not recorded

[1] The presence of Magnaporthe oryzae in each region is determined by the spread of the pathogen, which can be either localized or widespread. The information provided in this table is adapted from the **CABI Compendium** on *Magnaporthe oryzae* **(rice blast disease)**.

Supplementary materials_TS2

Table S2. Distribution of major, minor, and cloned *R* genes to blast among the 12 rice chromosomes.

**Chromosome N°**	**Major *R* genes**	**Minor *R* genes**	**Cloned *R* genes**	**Total *R* genes***
1	3	2	5	10
2	7	2	1	10
3	0	1	0	1
4	3	2	3	8
5	1	1	0	2
6	13	1	9	23
7	1	0	0	1
8	3	2	1	6
9	2	0	3	5
10	1	1	0	2
11	14	2	13	29
12	18	3	4	25
**Total**	66	17	39	122

* The distribution of blast resistance genes spans all 12 rice chromosomes, with the majority located on chromosomes 6, 11, and 12, harboring 23, 29, and 25 resistance genes, respectively. Chromosomes 3 and 7 contain the fewest blast resistance genes, with only one reported gene each, Pi66(t) and Pi17(t), respectively. Further details about these genes and their roles can be found within the text and in the cited references.
